# A large odontogenic myxoma of the bilateral maxillae: A case report

**DOI:** 10.3892/ol.2014.2243

**Published:** 2014-06-12

**Authors:** YING LIU, BO HAN, TAO YU, LONGJIANG LI

**Affiliations:** 1 State Key Laboratory of Oral Diseases, West China Hospital of Stomatology, Sichuan University, Sichuan, P.R. China; 2 Department of Head and Neck Oncology Surgery, West China Hospital of Stomatology, Sichuan University, Sichuan, P.R. China; 3 Department of Head and Neck Oncology, Sichuan Cancer Hospital, Chengdu, Sichuan, P.R. China

**Keywords:** odontogenic myxoma, bilateral maxillae, large tumor, radical maxillectomy, reconstruction

## Abstract

Odontogenic myxomas (OMs) are benign mesenchymal locally aggressive neoplasms of the jaw bone. Although OMs predominantly involve the mandible, maxillary tumors are usually more aggressive than mandibular tumors. The present study describes the case of a 37-year-old male with a large odontogenic myxoma of the bilateral maxillae, which caused a defect in the right skull base bone. The tumor was successfully removed through radical resection of the hard tissue and local resection around the envelope of the soft tissue. The tumor exhibited no recurrence. However, the current methods for bilateral maxillary reconstruction to restore the maxillary buttress and achieve an optimal aesthetic appearance are complicated due to the lack of suitable conditions for oral rehabilitation with good dentition.

## Introduction

Odontogenic myxomas (OMs) are benign, slow-growing, locally aggressive and non-metastasizing neoplasms of the jaw bone. OM is derived from embryonic mesenchymal elements of the dental anlage (1–3). According to the World Health Organization, OM is a benign tumor of ectomesenchymal origin with or without odontogenic epithelium (4). OM accounts for 0.5–20% of all odontogenic tumors (5,6). OM often occurs in individuals who are between their second and forth decades, has a slight predilection for females and is rarely found in children and the elderly (3). The majority of lesions that are without pain reach a large size and cause displacement of the teeth and asymmetry of the mandible or maxilla prior to discovery (7).

OM of the maxilla was first reported by Thoma and Goldman in 1947 (8). While OMs predominantly involve the mandible, maxillary tumors are more aggressive than those in the mandible (9). Certain lesions spread with progressive pain through the maxillary sinus and nasal cavity, and severe cases result in exopthalmus, nasal obstruction and neurological disturbance (7,10). The surgical treatment of OM, including curettage and radical resection, is controversial due to the varying recurrence rates (9,11).

The present study reports the case of a male patient with a large maxillary OM. Although the mass was removed successfully, repairing the defect in the maxilla was a complex process. Patient provided written informed consent.

## Case report

### Patient presentation

**Figure 1:**
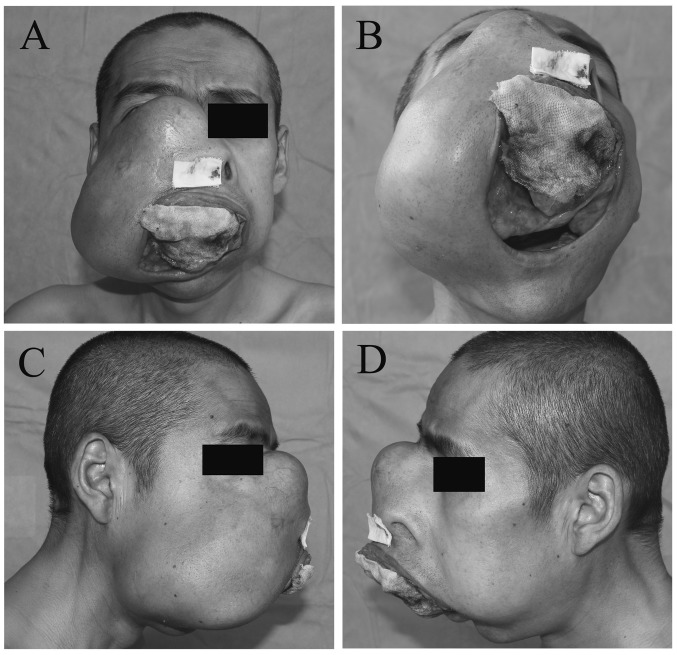
Patient with a large maxillary odontogenic myxoma. (A) The tumor occupied the bilateral maxillae and the nose, extending superoinferiorly from the right orbit and infraorbital rim to the bilateral alveolar bone and hard palate. (B) The tumor filled the oral cavity, causing partial displacement of the teeth and subsequent protrusion outward from the mouth with rough surface and exuded pus on palpation. (C and D) The infraorbital rim disappeared and the right eyeball was pushed up by the tumor.

A 37-year-old male presented with a mass on the right side of the face, which had persisted for five years, with accelerated growth for one year, causing serious facial deformity and difficulty in eating. The painless mass was ~16×16 cm and occupied the right maxilla and the nose, extending superoinferiorly from the right orbit and infraorbital rim to the bilateral alveolar bone and hard palate. Part of the mass displaced the teeth and protruded outward from the mouth, with a rough surface that released a purulent discharge upon palpation. No superficial ulceration, sinuses or fistulas were observed on the overlying skin, which did not adhere to the mass. The patient had bilateral nasal obstruction with effluvial secretion and normal vision, even though the infraorbital rim disappeared, as the mass displaced the optic nerve (Fig. 1).

### Clinical and imaging analyses

**Figure 2:**
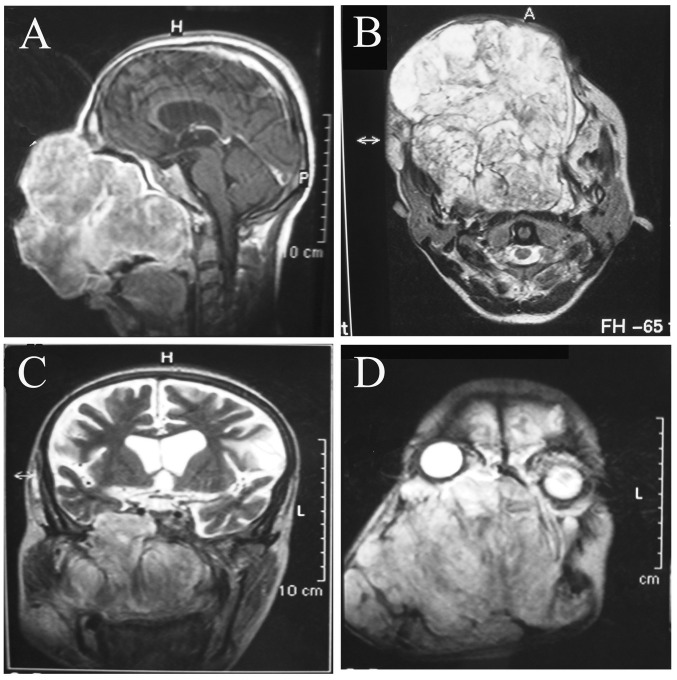
Patient with a large odontogenic myxoma of the partial left maxilla and whole right maxilla. (A) Sagittal T1-weighted MRI scan. (B) Axial T2-weighted MRI scan. (C and D) Coronal T2-weighted MRI scan revealing that the partial right sphenoid wing, the bilateral maxillary sinus and the nasal septum were absorbed by the tumor pressure. MRI, magnetic resonance imaging.

Enhanced computed tomography revealed that an irregular and low-density shadow without obvious enhancement was displaced in the maxillae, and that a high-density shadow was interspersed in it. Magnetic resonance imaging identified that the well-circumscribed tumor had already obliterated the maxillae, the maxillary process of the right zygomatic bone, part of the left maxilla, the ethmoidal sinuses, the sphenoid sinus and the nasal cavity. The nasopharyngeal cavity and the left maxillary sinus were observed to be narrow, and the turbinate bones and the nasal septum had been resorbed due to the tumor pressure. Although the large mass grew into the intracranial cavity through a bone defect in the sphenoid wing caused by tumor pressure-induced resorption, the tumor was present with well-defined borders under the dura mater (Fig. 2). According to the clinical and imaging examinations, it was hypothesized that the tumor may be a mesenchymal benign tumor with malignant transformation.

### Surgery

**Figure 3:**
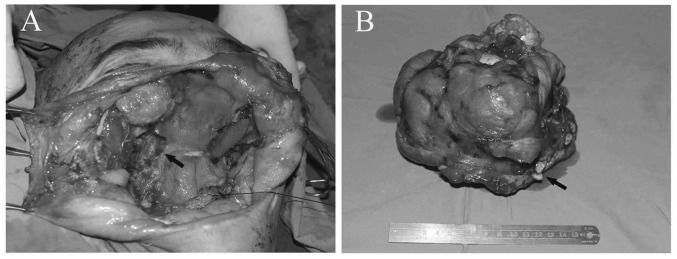
Macroscopic images of the surgical procedure. (A) Intraoperative image showing that following tumor removal, the cavernous sinus (black arrow) was exposed and the nasal septum was pushed to the left. (B) Odontogenic myxoma with tooth displacement (black arrow).

Following a tracheostomy, the patient underwent right radical maxillectomy and left partial maxillectomy, which included the partial right zygomatic bone and zygomatic alveolar ridge of the left maxilla, with a 1-cm healthy margin. Moreover, the mass was enucleated from the soft tissues around its capsule. A small quantity of bone surrounding the bone defect in the skull base, as well as the turbinate mucosa and the thin skin covering the mass were also resected. The medial rectus in the right eye, the septum mucosa, the left nasal mucosa, the thin bone of the left infraorbital rim and the residual posterior wall of the maxillary sinus were retained. Subsequent to tumor removal, the cavernous, ethmoid and left maxillary sinuses were exposed (Fig. 3). The patient did not undergo repair using soft-tissue flaps due to the risk of complications and financial constraints. Thus, the wound surface was covered with Heal-All Rehabilitation Membrane^®^ (heterogeneous acellular dermal matrix of cattle; Zhenghai Biological Co., Ltd., Shandong, China) and the defect cavity was filled with large numbers of staple slivers with iodoform. No intracranial infection or cerebrospinal leakage were observed. No clinical or radiographical signs of recurrence were observed, and the soft- and hard-tissue defects were covered with compact mucosa after one year of post-operative control. The right side of the patient’s face and nasal bridge collapsed and the right eyeball moved down, as the defects were not repaired using soft flaps.

### Diagnosis

Macroscopically, the surgically resected mass measured ~15×16×16 cm and appeared as a completely encapsulated whitish-grey, lobulated, smooth and hard mass. Microscopically, the tumor was composed of a faintly basophilic myxomatous ground substance and a mount of spindle- and stellate-shaped cells. Variable quantities of fibrous tissues were found throughout the mucoid-rich matrix. Furthermore, minimal and inconspicuous thin-walled vessels and residual bone fragments were interspersed within the tumor. Islands of odontogenic epithelium, hyalinization and calcification were not found. The mass was diagnosed as an OM.

## Discussion

An OM is a mesenchymal benign tumor with the potential for extensive bone destruction, cortical expansion and a relatively high recurrence rate (8,12). It has been found that OM accounts for ~7.2% of all odontogenic tumors and has no marked predilection in terms of gender and location (mandible or maxilla) in the Chinese population (12,13). By contrast, other studies have reported a male:female ratio of between 1:2 and 1:3 and a mandible:maxilla ratio of between 2:1 and 2.5:1, particularly in African countries (12,13). OM has been reported in individuals of a wide range of ages, with an average age of 25.3 years old. A total of eight cases <10 years old and none >60 years old have been reported at the West China Hospital of Stomatology (Chengdu, China) (13). The majority of patients with OM present with a slowly increasing swelling and facial asymmetry with infiltration. The mass is locally aggressive, particularly in the maxilla (4,14). In the present case, the growth of the mass had accelerated for one year prior to surgery due to the accumulation of mucoid ground substance. Moreover, the tumor contained residual bone fragments and nasal mucosa with tumor infiltration.

While surgical intervention is recommended, no consensus has been reached with regard to the surgical method for the treatment of OM due to its aggressive nature and high rate of recurrence, which is between 10 and 43%, particularly in patients who are treated with curettage or local surgical excision (15). Boffano *et al* (11) reported that conservative surgery, such as curettage and enucleation, should be performed when tumors are <3 cm, while radical surgery should be performed when tumors are large and aggressive (11,16,17). Locally aggressive OM should be subjected to radical resection with a margin of 0.5–1.0 cm or 1.0–1.5 cm of healthy bone (11,18). In the present case, based on the absorption of the bilateral maxillae and the partial right sphenoid wing, an extended resection of the mass was performed, with 1 cm margins in the right zygomatic bone and the zygomatic alveolar ridge of the left maxilla to prevent recurrence. The tumor was enucleated around its complete envelope in the soft tissue. Moreover, the thin skin covering the mass was resected and was not found to be infiltrated in the frozen histopathology report. A partial or radical resection was hypothesized to be the best treatment choice for maxillary OM, rather than conservative surgery, in order to reduce the recurrence rate.

**Figure 4:**
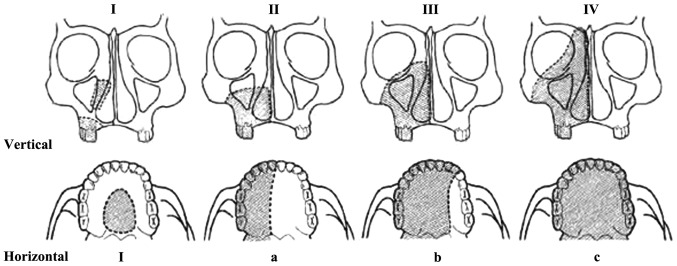
Brown’s classification system for maxillary defects (24). Vertical component; Classes I, maxillectomy with no oroantral fistula; II, low maxillectomy III, high maxillectomy; and IV, radical maxillectomy. Horizontal component: I, unilateral alveolar maxilla and resection of the hard palate; a, resection of less than or equal to half of the alveolar and hard palate, not involving the nasal septum or crossing the midline; b, resection of the bilateral alveolar maxilla and hard palate, including a smaller resection that crosses the midline of the alveolar bone, including the nasal septum; and c, removal of the entire alveolar maxilla and hard palate.

The development of microsurgery and reconstructive surgery techniques have enabled the basic functional and aesthetic aims of maxillary reconstruction to be achieved. Various methods have been used to reconstruct maxillary defects, including vascularized soft- or hard-tissue grafts from the radial forearm, rectus abdominis, anterolater thigh, scapular and fibular, iliac crest flaps, vascularized free fibular flaps and cranial bone flaps, as well as material implants using, for example, titanium mesh (19–23). According to the Brown classification system for maxillary defects (24), the patient described in the present study had defects higher than class IV (Fig. 4), with the large soft- and hard-tissue defects being difficult to reconstruct. In this case, the residual thinning bones and soft tissue had insufficient strength to support the weight of a composite bone flap with titanium miniplates. Peng *et al* (20) reported that the composite fibular flap is inadequate for restoring the maxilla and the infraorbital area. Smolka and Iizuka (25) used a latissimus dorsi flap/rectus abdominis flap and free iliac crest graft to repair a class IVa defect with the highest rate of transplant loss. Although vascularized soft-tissue flaps are not capable of restoring the maxillary buttress, they have been reported to fill the partial large cavity of midfacial defects and rehabilitate the basic swallowing function of patients. In the present case, the use of a latissimus dorsi myocutaneous flap and an anterolateral femoral skin flap was proposed for the repair of the skull base bone and maxillary defects in order to alleviate the symptoms of right facial collapse. Secondary reconstruction with a prosthesis was also proposed. However, the patient refused aesthetic reconstruction.

To the best of our knowledge, the present study is the first report of such a large maxillary OM. In the present study, without surgical intervention, the patient had a high risk of succumbing due to increasing intracranial pressure. The mass was removed successfully without any complications and did not recur within one year despite the unsatisfactory results with regard to the external facial features and their function. Due to the structure of the maxillae and the local infiltration of OM, radical resection may the best treatment choice for maxillary OM in hard tissue and local resection may be best around the envelope in soft tissue. It is difficult to restore the maxillary buttress and achieve an optimal aesthetic appearance using current methods for reconstructing midfacial defects due to the lack of suitable conditions for oral rehabilitation with good dentition. Further investigations are required to develop appropriate, satisfactory reconstruction methods for midfacial defects.
